# Biomarkers in the diagnostic algorithm of myalgic encephalomyelitis/chronic fatigue syndrome

**DOI:** 10.3389/fimmu.2022.928945

**Published:** 2022-10-10

**Authors:** Sabine Gravelsina, Anda Vilmane, Simons Svirskis, Santa Rasa-Dzelzkaleja, Zaiga Nora-Krukle, Katrine Vecvagare, Angelika Krumina, Iana Leineman, Yehuda Shoenfeld, Modra Murovska

**Affiliations:** ^1^ Institute of Microbiology and Virology, Rīga Stradiņš University, Riga, Latvia; ^2^ Department of Infectology, Rīga Stradiņš University, Riga, Latvia; ^3^ Zabludowicz Center for Autoimmune Diseases, Sheba Medical Center, Affiliated with the Sackler Faculty of Medicine, Tel-Aviv University, Tel Aviv, Israel

**Keywords:** ME/CFS, β2AdR antibodies, AChR antibodies, HHV-6, biomarkers

## Abstract

Myalgic encephalomyelitis/chronic fatigue syndrome (ME/CFS) is a complex disease that is mainly diagnosed based on its clinical symptoms. Biomarkers that could facilitate the diagnosis of ME/CFS are not yet available; therefore, reliable and clinically useful disease indicators are of high importance. The aim of this work was to analyze the association between ME/CFS clinical course severity, presence of HHV-6A/B infection markers, and plasma levels of autoantibodies against adrenergic and muscarinic acetylcholine receptors. A total of 134 patients with ME/CFS and 33 healthy controls were analyzed for the presence of HHV-6A/B using PCRs, and antibodies against beta2-adrenergic receptors (β2AdR) and muscarinic acetylcholine receptors (M3 AChR and M4 AChR) using ELISAs. HHV-6A/B U3 genomic sequence in whole-blood DNA was detected in 19/31 patients with severe ME/CFS, in 18/73 moderate ME/CFS cases, and in 7/30 mild ME/CFS cases. Severity-related differences were found among those with a virus load of more than 1,000 copies/10^6^ PBMCs. Although no disease severity-related differences in anti-β2AdR levels were observed in ME/CFS patients, the median concentration of these antibodies in plasma samples of ME/CFS patients was 1.4 ng/ml, while in healthy controls, it was 0.81 ng/ml, with a statistically significant increased level in those with ME/CFS (*p* = 0.0103). A significant difference of antibodies against M4 AChR median concentration was found between ME/CFS patients (8.15 ng/ml) and healthy controls (6.45 ng/ml) (*p* = 0.0250). The levels of anti-M4 plotted against disease severity did not show any difference; however, increased viral load correlates with the increase in anti-M4 level. ME/CFS patients with high HHV-6 load have a more severe course of the disease, thus confirming that the severity of the disease depends on the viral load—the course of the disease is more severe with a higher viral load. An increase in anti-M4 AchR and anti-β2AdR levels is detected in all ME/CFS patient groups in comparison to the control group not depending on ME/CFS clinical course severity. However, the increase in HHV-6 load correlates with the increase in anti-M4 level, and the increase in anti-M4 level, in turn, is associated with the increase in anti-β2AdR level. Elevated levels of antibodies against β2AdR and M4 receptors in ME/CFS patients support their usage as clinical biomarkers in the diagnostic algorithm of ME/CFS.

## Introduction

Myalgic encephalomyelitis/chronic fatigue syndrome (ME/CFS) is a severe disease that occurs in all ethnic and racial groups in countries around the world with an estimated prevalence of 0.1%–2.2% depending on disease criteria used (1, 2). Although beliefs that ME/CFS could be imaginary have been eradicated long ago, the exact pathogenesis of ME/CFS is still unknown. One hypothesis is that it is a complex multifactorial syndrome in which immunological and environmental factors play an essential role. The lack of progress in ME/CFS research has been attributed to controversy around diagnosis, because there are no approved laboratory tests. The diagnosis of ME/CFS is based mainly on clinical symptoms—severe fatigue that cannot be relieved by rest, post-exertional malaise, cognitive impairment, autonomic dysfunction, exhaustion, sensitivity to light, and unexplained pain (3). Biomarkers that could facilitate the diagnosis of ME/CFS are not yet available; therefore, reliable and clinically useful disease indicators are of a high importance.

ME/CFS disease onset is often reported to be triggered by infections, and the link between infections and autoimmune diseases is well established (4–6). In our previous studies, we showed that HHV-6 is implicated in the development of encephalopathy and ME/CFS (7, 8). Immunologic disturbance associated with ME/CFS may be the result of viral infection or may lead to reactivation of latent viruses. Once reactivated, the viruses may contribute to the morbidity of ME/CFS *via* inflammation and immune dysregulation, especially the herpesviruses EBV and HHV-6, which infect immune cells. Viral infections can trigger an autoimmune response as well. In the majority of ME/CFS cases, there is no conclusive evidence for chronic viral infection, but it is plausible that viruses could act *via* a “hit and run” mechanism; this theory proposes that viruses trigger the disease, cause immune abnormalities, and leave a dysfunctional immune system and/or autoimmunity. Infection-triggered disease onset, chronic immune activation, and autonomic dysregulation in ME/CFS point to an autoimmune disease involving neurotransmitter receptors. Autoantibodies against G-protein-coupled receptors were shown to play a pathogenic role in several autoimmune diseases.

A study published in 2020 shows the finding of elevated autoantibodies against beta2-adrenergic receptors (β2AdR) and muscarinic acetylcholine receptors (M3 AChR and M4 AChR) in some individuals with ME/CFS (9). Since both of those receptors are important in vasoconstriction of blood vessels, their functional disturbance would be expected to cause vasoconstriction and hypoxemia, which would explain many of the symptoms of ME/CFS (9). Another study published in the same year demonstrates the correlation between levels of β2AdR antibodies and brain network changes associated with pain (10).

We hypothesize that the cohort of ME/CFS patients is divided into a number of groups/subsets, one of which is a group/subset having primary autoimmune etiology, and another one is triggered by viral infection. The group/subset triggered by viral infection could also have markers of autoimmunity. It is possible to find a set of viral and autoimmunity markers pointing towards ME/CFS, especially later after disease onset. The aim of this work was to determine plasma levels of autoantibodies against adrenergic and muscarinic acetylcholine receptors in patients with ME/CFS and healthy controls, and to analyze the association between ME/CFS clinical course severity, the presence of HHV-6A/B infection markers, and plasma levels of autoantibodies against adrenergic and muscarinic acetylcholine receptors.

## Materials and methods

A total of 134 patients [42 men (23–76 years old) and 92 women (23–68 years old)] at the Rīga Stradiņš University Ambulance outpatient clinic were diagnosed with ME/CFS based on the Fukuda criteria (11). Out of 42 male patients, 10 had a severe, 26 had a moderate, and 6 had a mild disease course. Out of 92 female patients, 21 had a severe, 47 had a moderate, and 24 had a mild disease course. There were no other medical or neurological diseases that may cause fatigue. Thirty-three ethnically and age-matched, but not gender-matched (female:male = 1:4) healthy blood donors from the Latvia State Blood Donor center were included in the study as a control group [27 men (18–74 years old) and 6 women (20–72 years old)]. All patients were interviewed with questionnaires to evaluate various categories. To examine the symptom pattern in ME/CFS patients, we used adapted semi-structured interview questions created by Minnock et al. (12). A total of 27 questions were structured in six sections: causes and triggers of fatigue; character of fatigue; current symptoms; comorbidities; solutions for fatigue; and its influence on work disability. Multiple choice answers were provided for each question. Regarding sleep disturbances, we included a self-reported questionnaire—Athens Insomnia Scale 8 (13)—to assess insomnia symptoms, which included the evaluation in various sleep-related questions: sleep induction, awakenings during the night, final awakening, sleep quality, wellbeing during the day, functioning capacity during the day, and sleepiness during the day. We used the cutoff value of ≥6 points for the confirmation of sleep disturbances. VAS, ranging from 0 to 10, was also measured for all patients to assess the disease-related pain intensity. Based on the previously described questions in the questionnaire, points were given, and answers were graded defining the disease course as mild (0–7 points), moderate (8–12 points), or severe (13–15 points).

DNA from 134 patients’ whole-blood samples was isolated using the standard phenol–chloroform extraction method. The quantity and quality of the DNA was measured using a spectrophotometer and β-globin PCR was also performed (14). The extracted DNA (~200 ng/μl) was used as a template in nested polymerase chain reaction (nPCR), using primers amplifying the HHV-6 A/B U3 gene sequence as described previously (15). Used primers were complementary to the gene that encodes main capsid proteins for both HHV-6A and HHV-6B. HHV-6 genomic DNA (Advanced Biotechnologies Inc, Columbia, MD, USA) was used as a positive control. The sensitivity of HHV-6-specific primers was three copies per reaction (16). HHV-6A and HHV-6B were differentiated according to the methodology described by Lyall and Cubie (17). A two-step PCR reaction was performed again to differentiate between the two subtypes of the virus with primers targeting the HHV-6 large tegument protein (LTP) gene following *Hind*III restriction analysis. The obtained nPCR amplicons were digested with *Hind*III restriction endonuclease (Thermo Scientific, Waltham, MA, USA) according to the manufacturer’s protocol. *Hind*III cleaves the HHV-6B-positive sample into two fragments of 66 bp (base pair) and 97 bp, but does not cleave HHV-6A at all. All PCR results were visualized using 1.7% agarose electrophoreses gel. Viral load was detected using HHV-6 Real-TM Quant Real-TM kit from Sacace Biotechnologies (Como, Italy). The viral load was calculated as copies per 10^6^ peripheral blood mononuclear cells (copies/10^6^ PBMCs). Plasma samples of patients and healthy controls were analyzed for anti-muscarinic cholinergic receptor 3 (M3) and receptor 4 (M4) autoantibodies using ELISAs according to the manufacturer protocols from CellTrend, GmbH (Luckenwalde, Germany). Due to the insufficient volume of blood plasma sample, only 67 out of the previously described 134 ME/CFS patients were analyzed for antibodies against beta 2 adrenergic receptor (β2AdR) using the validated human adrenergic beta-2 receptor surface (β2AdR) quantitative ELISA kit from BlueGene (Shanghai, China). The study design was approved by the Ethical Committee of Rīga Stradiņš University (Ethical code Nr.6-1/05/33 and date of approval 30 April 2020) and a written consent was obtained from all participants.

Statistical analysis was performed using GraphPad Prism v.9.0.2 software for Mac (San Diego, CA, USA). To compare the proportions of respective cases between the groups, the Chi-square (*χ* ^2^) test was applied. The normality of the numerical data was determined by D’Agostino and Pearson, and Shapiro–Wilk normality tests, and to compare between-group differences, nonparametric Mann–Whitney *U* test or Kruskal–Wallis variance analysis followed by the two-stage linear step-up procedure of Benjamini, Krieger, and Yekutieli were used. To check usefulness of the detection of antibodies to M3, M4 cholinergic, and β2 adrenergic receptors to discriminate ME/CFS patients from healthy individuals, ROC analysis was done. To determine the relationship between the levels of antibodies to cholinergic and adrenergic receptors, the disease severity, and HHV-6 load, Spearman’s rank correlation analysis was performed. The mean levels of the parameters were expressed as medians with dispersion, characterized by the interquartile range (IQR), and statistical significance was set at *p* < 0.05.

## Results

**Figure 1:**
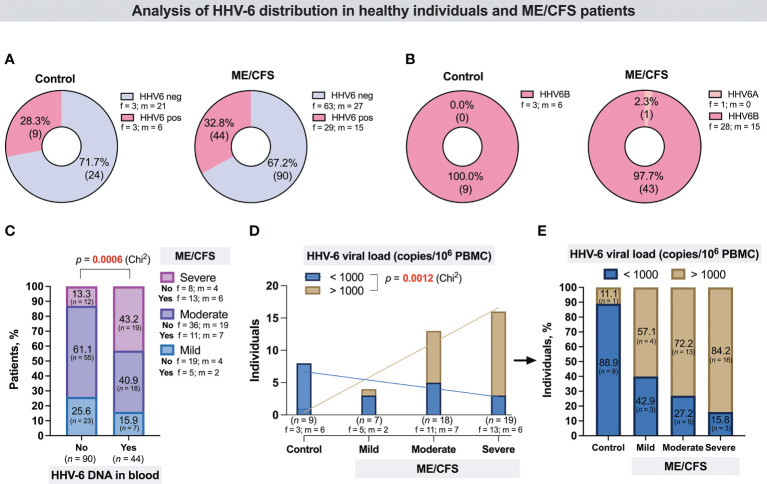
**(A)** Proportion of HHV-6-positive and -negative individuals in the control group and the ME/CFS patient group. **(B)** Proportion of individuals in the control group and the ME/CFS patient group with detected HHV-6A and HHV-6B. **(C)** Proportion of HHV-6-positive and -negative patients with different ME/CFS severity; “no”—without HHV-6 genomic sequence in blood DNA, “yes”—with HHV-6 genomic sequence in blood DNA. **(D)** Number of control individuals and of mild, moderate, and severe ME/CFS disease course patients with viral load < 1,000 copies/10^6^ PBMCs and > 1,000 copies/10^6^ PBMCs. **(E)** Proportion of control individuals and ME/CFS patients with viral load < 1,000 copies/10^6^ PBMCs and > 1,000 copies/10^6^ PBMCs. f, ,female; m, male.

**Figure 2:**
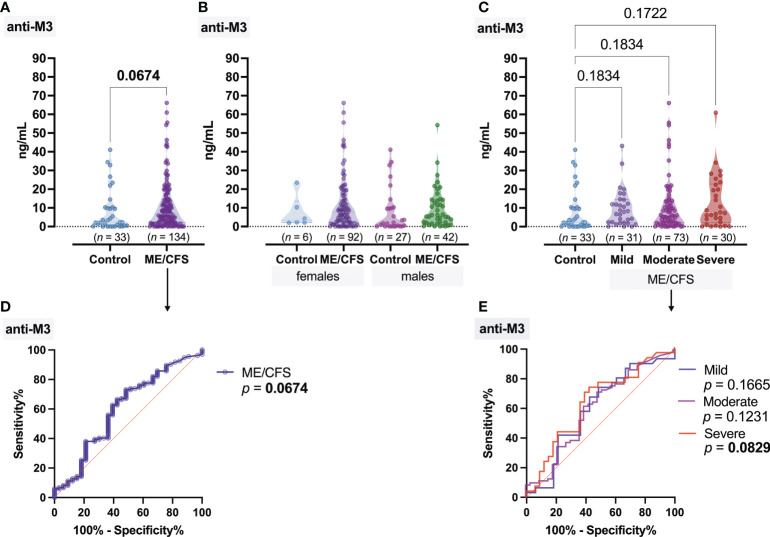
Concentration of antibodies to muscarinic cholinergic receptor 3 (anti-M3): **(A)** Healthy control (donors) and ME/CFS patients. **(B)** Regarding women and men of both groups. **(C)** Regarding ME/CFS severity; numbers above graphs show *p*-value, determined by Mann–Whitney test **(A)** or Kruskal–Wallis test with respective post-test **(B, C)**. **(D, E)** ROC analysis showing the potential of anti-M3 as a discriminating marker for ME/CFS.

From all the cohort, HHV-6A/B U3 genomic sequence in whole-blood DNA was detected in 9/33 (28.3%) control group individuals and in 44/134 (32.8%) patients with ME/CFS (**Figure 1A**), including 19/31 (61.29%) patients with severe, 18/73 (24.66%) with moderate, and 7/30 (23.33%) with mild ME/CFS. Proportion analysis of HHV-6A/B-positive and -negative patients between groups showed that HHV-6A/B are more frequent among patients with severe ME/CFS (43%) compared to patients with mild ME/CFS (16%) (*p* = 0.0006) (**Figure 1B**). In all ME/CFS cases except one (HHV-6A), the presence of HHV-6B was confirmed. All control group individuals were HHV-6B positive. The results showed that the median HHV-6 load in whole blood from patients with mild, moderate, and severe ME/CFS disease was 4,138 copies/10^6^ PBMCs (IQR: 19–8,244), 7,302 copies/10^6^ PBMCs (IQR: 668–15,045), and 5,498 copies/10^6^ PBMCs (IQR: 1,158–9,048), respectively, and no severity-related differences were found among these patients. Nevertheless, severity-related differences were found among those with a viral load of more than 1,000 copies/10^6^ PBMCs. Proportion analysis showed that 84.2% of ME/CFS patients with severe clinical course, 72.2% with moderate clinical course, and 57.1% with mild clinical course had a viral load of >1,000 copies/10^6^ PBMCs in comparison to the control group where only one of HHV-6-positive individuals (11.1%) had a viral load of >1,000 copies/10^6^ PBMCs (**Figures 1C–E**). The median concentration of anti-muscarinic cholinergic receptor 3 (anti-M3) antibodies in ME/CFS patients’ plasma samples was 6.75 ng/ml, while in healthy donors, it was 2.29 ng/ml, without a significant difference between cases of ME/CFS and healthy controls (**Figure 2A**), as well as between female and male patients (**Figure 2B**), and without significant differences regarding disease severity (**Figure 2C**). ROC analysis did not reveal anti-M3 as a discriminating marker for ME/CFC (**Figures 2D, E**).

**Figure 3:**
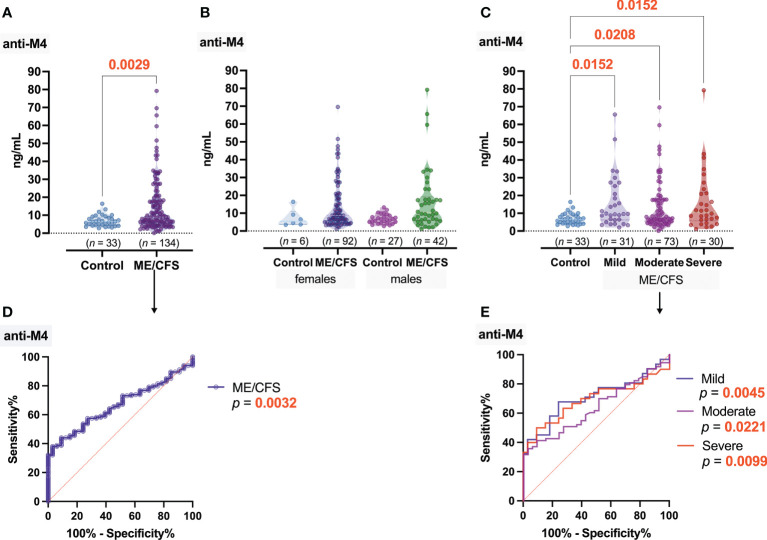
Concentration of antibodies to muscarinic cholinergic receptor 4 (anti-M4): **(A)** Healthy control (donors) and ME/CFS patients. **(B)** Regarding women and men of both groups. **(C)** Regarding ME/CFS severity; numbers above graphs show *p*-value, determined by Mann–Whitney test **(A)** or Kruskal–Wallis test with respective post-test **(B, C)**. **(D, E)** ROC analysis showing the potential of anti-M4 as a discriminating marker for ME/CFS.

Significant differences of anti-muscarinic cholinergic receptor 4 (anti-M4) antibody concentration was found between ME/CFS patients and healthy donors (*p* = 0.0029); the median concentration of anti-M4 in ME/CFS patients’ plasma samples was 8.95 ng/ml, while that in healthy donors was 6.45 ng/ml (**Figure 3A**). Anti-M4 levels did not differ significantly between the ME/CFS female patients and women from the donor group; however, significant differences (*p* = 0.0199) were found between the male groups (**Figure 3B**). Regarding the severity of disease, the median levels of anti-M4 (**Figure 3C**) were almost the same in all three subgroups of patients—9.64, 8.00, and 10.62 ng/ml for mild, moderate, and severe ME/CFS, respectively. ROC analysis showed that, in contrast to anti-M3, anti-M4 could serve as a good marker for ME/CFS in general (**Figure 3D**); however, it could not serve as a discriminating factor regarding ME/CFS severity (**Figure 3E**).

**Figure 4:**
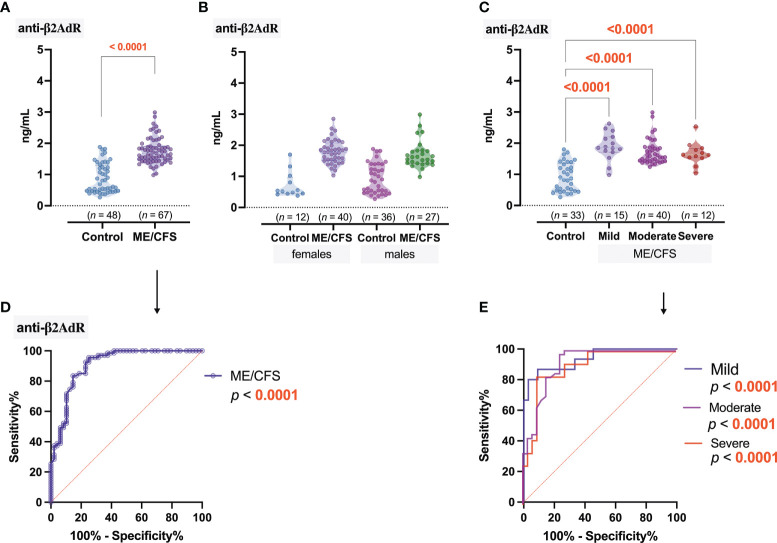
Concentration of antibodies to β2 adrenergic receptors (anti-β2AdR): **(A)** Healthy control (donors) and ME/CFS patients. **(B)** Regarding women and men of both groups. **(C)** Regarding ME/CFS severity; numbers above graphs show *p*-value, determined by Mann–Whitney test **(A)** or Kruskal–Wallis test with respective post-test **(B, C)**. **(D, E)** ROC analysis showing the potential of anti-β2AdR as a discriminating marker for ME/CFS.

A significant (*p* < 0.0001) increase in anti-β2AdR levels was observed in ME/CFS patients (1.4 ng/ml) compared to healthy controls (0.81 ng/ml) (**Figure 4A**), and these statistical differences were also found in the respective female and male groups (**Figure 4B**). However, no differences in disease severity-related anti-β2AdR levels were observed in ME/CFS patients (**Figure 4C**). The ROC analysis showed that, in this study, anti-β2AdR has the best potential to be a good marker of ME/CFS in general (**Figure 4D**), but, as with anti-M4, cannot be a discriminating factor for the severity of ME/CFS (**Figure 4E**).

**Figure 5:**
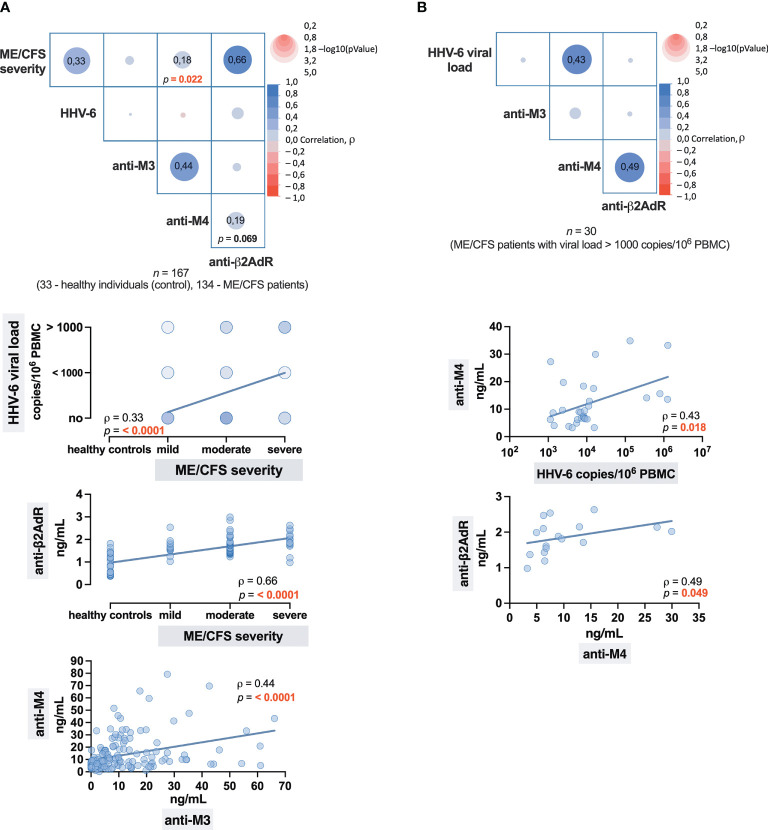
Correlation matrices with correlation graphs representing the strength of the association: **(A)** Between the ME/CFS severity and HHV-6 infection, and levels of antibodies to cholinergic M3 (anti-M3), M4 (anti-M4), and β2 adrenergic (anti-β2AdR) receptors. **(B)** Between the levels of HHV-6 viral load and anti-M3, anti-M4, and anti-β2AdR. The numbers in cells (squares) show the value of the Spearman’s rank correlation coefficient rho (ρ).

Initially, Spearman’s rank correlation analysis was performed, including all subjects—33 healthy donors and 134 patients with ME/CFS, and found a positive association (*ρ* = 0.33) between the severity of ME/CFS and HHV-6 viral load, as well as between severity of ME/CFS and anti-β2AdR level (*ρ* = 0.66) and, in a lesser extent, anti-M4 (*ρ* = 0.18). A positive relationship between anti-M3 and anti-M4 (*ρ* = 0.44) was also found (**Figure 5A**).

To more accurately determine the relationship between levels of HHV-6 viral load and antibody levels in ME/CFS patients, the following correlation analysis was performed, and the results (**Figure 5B**) showed a positive relationship between HHV-6 copy number and anti-M4 level (*ρ* = 0,43), as well as between anti-M4 and anti-β2AdR (ρ = 0.49). Therefore, anti-M4 and anti-β2AdR can be assumed as double markers for ME/CFS, where the level of anti-M4 may also indicate the severity of HHV-6 infection as well—higher anti-M4 levels are well associated with higher viral load.

## Discussion

With an estimated prevalence of 0.1%–2.2%, ME/CFS is a multisystem disease with not yet established etiology. Patients suffer from persistent exhaustion, cognitive impairment, autonomic dysfunction, chronic pain, and flu-like symptoms, leading to a substantial reduction of life quality (18). Clinical heterogeneity in disease onset (infection versus non-infection triggered), the presence of immune-associated symptoms, and divergent immunological alterations point to the existence of subgroups of ME/CFS patients with possibly different pathomechanisms. Therefore, it is important to identify clinically useful diagnostic markers to stratify the patients into subgroups to ensure the selection of more appropriate treatment for each subgroup.

HHV-6 has long been considered as one of the ME/CFS trigger factors, and recent published data indicate that HHV6-B is associated with ME/CFS disease severity (19). The high HHV-6 load, as well as infection in active phase, has been described in patients with fibromyalgia as well (20). According to literature sources, HHV-6 A/B may persist in many cells and tissues long after it has disappeared from the circulation and therefore herpes viruses have been repeatedly hypothesized to underlie the chronic relapsing/remitting form of ME/CFS due to their persistence in a latent form with periodic reactivation. There are data that show that HHV-6 reactivation causes symptoms of ME/CFS such as fatigue and cognitive and autonomic dysfunction (21). Our study shows that HHV-6 persistent infection is more frequently detected among patients with severe ME/CFS than those with a mild disease course. Moreover, according to the proportion analysis, the highest percentage of ME/CFS patients with high viral load (>1,000 copies/10^6^ PBMCs) is estimated in the group with severe clinical course (84.2%), followed by groups with moderate (72.2%) and mild (57.1%) disease course, thus confirming that the severity of the disease depends on the viral load—the course of the disease is more severe with a higher viral load. A limitation of this study is that only HHV-6 U3, a late gene, was examined, which would not be expressed in the case of abortive lytic replication in which early gene expression takes place, but not late gene expression, and no new virus is produced.

In many studies, ME/CFS has been linked to viral or bacterial infections. Despite the fact that one causative agent has not been indicated, the link between infections and autoimmune diseases is well established (4, 6). Although the exact ME/CFS pathogenesis is still unknown, the most plausible hypothesis is that dysregulation of the immune system, the autonomic nervous system, and metabolic disturbances contribute to this complex syndrome, in which severe fatigue and cognitive impairment are a central feature. Autonomic dysregulation, chronic immune activation, and infection-triggered disease onset point to an autoimmune disease involving neurotransmitter receptors. Autoantibodies against G-protein-coupled receptors are involved in the normal physiological regulation of cardiovascular, respiratory, and gastrointestinal systems and even in the regulation of the immune system (22). According to the autoimmune concept in genetically predisposed patients, triggers such as a virus can lead to hyperstimulation of B cells. The appearance of higher anti-GPCR antibody titers becomes a cause of the dysregulation of the autonomic nervous system. Such autoimmune dysautonomia may be the possible explanation of a wide spectrum of symptoms that are described not only in patients with ME/CFS but also in those with post-COVID-19 syndrome, fibromyalgia, postural orthostatic tachycardia syndrome, and rheumatic diseases (23).

Bynke et al. reported significant increases in autoantibody levels against M3/M4 AchR, β1AdR, and β2AdR in ME/CFS patients compared to controls, but no significant correlations were found between autoantibody levels and disease severity (24). Our study also shows a significant increase in anti-M4 and anti-β2AdR levels in the ME/CFS patients’ group in comparison to healthy controls. Moreover, the increase in anti-M4 level correlates with the increase in HHV-6 load, and the increase in anti-M4 level in turn is associated with the increase in anti-β2AdR level. The increase in levels of anti-M4 AchR and anti-β2AdR can be found in mild, moderate, and severe ME/CFS patients compared to controls, but no significant correlations were found between autoantibody levels and disease severity. One of the disadvantages of this study is a different gender matching proportion between ME/CFS patients and healthy controls since only 6 of the 33 control subjects were female. Despite the fact that there is a statistically significant difference in anti-M4 AchR level between the 134 patients and the 33 donors (*p* = 0.0029), anti-M4 levels do not differ significantly between women in ME/CFS (92) and the control group (6) (*p* = 0.2358), indicating that sample size in the control group might not be enough to draw accurate conclusions. However, a significant difference (*p* = 0.0199) is found between male ME/CFS patients (42) and the control group (25) when the ratio was near to 1:1. No differences in levels of antibodies against β2AdR, M3 AchR, or M4 AChR were observed between women and men, either in the ME/CFS patient group or in the control group.

It is shown that the removal of immunoglobulins and immune complexes including anti-β2 AdR and anti-M3/M4 AchR antibodies during immunoadsorption leads to a rapid symptom improvement in most patients with ME/CFS (26), and the reduction of pre-treatment elevated β2AdR autoantibody titers is demonstrated by Loebel et al. in clinical responders to B-depletion therapy by rituximab (25).

Antibodies against β2AdR and M3/M4 AchR are significantly elevated in ME/CFS patients compared to controls (25). It is shown that 29.5% of patients with ME/CFS have elevated antibodies against one or more M AChR and β2AdR. Although the function of these antibodies in ME/CFS at present is unclear, the association of β2AdR and M AChR antibodies with immune activation markers may support a pathogenic role and warrants their testing as potential biomarkers in clinical trials. The association of autoantibodies with immune markers suggests that they activate B and T cells expressing β2AdR and M AChR (25). Dysregulation of acetylcholine and adrenergic signaling could also explain various clinical symptoms of ME/CFS.

Freitag et al. demonstrate the connection between anti-GPCR antibodies and severity of key clinical symptoms in ME/CFS. Interestingly, only patients with infection-triggered onset titers of antibodies against α 1/2 AdR, α 1/2/3 AdR, and M3/4 AchR correlate with the severity of fatigue, muscle pain, and cognitive symptoms (27). Anti-α1/2 AdR antibodies are found in correlation with gastrointestinal symptoms, and anti-α1-, β2/3-AdR, and M4-AChR antibodies are found in correlation with pupillomotor symptoms (27). In contrast, the non-infection triggered onset group shows only strong correlations of anti-α1/2-AdR and β1/2/3-AdR antibodies with orthostatic dysfunction, which, also with anti-M4 AchR antibodies, are described in patients with postural orthostatic tachycardia syndrome (28).

Identification of β2AdR and AChR antibodies in ME/CFS patients provides a strong indication that, at least in a subset of ME/CFS patients, the disease has an autoimmune etiology. It is conceivable that various symptoms of ME/CFS including cognitive deficits, autonomic dysregulation, and immune activation could be partly mediated by autoantibodies against these receptors in a subset of patients.

## Conclusions

The obtained results show that ME/CFS patients with high HHV-6 load have a more severe course of the disease, thus confirming that the severity of the disease depends on the viral load—the course of the disease is more severe with a higher viral load.

An increase in anti-M4 AchR and anti-β2AdR levels is detected in all ME/CFS patient groups in comparison to the control group not depending on ME/CFS clinical course severity. However, the increase in HHV-6 load correlates with the increase in anti-M4 level, and the increase in anti-M4 level, in turn, is associated with the increase in anti-β2AdR level.

Elevated levels of antibodies against β2AdR and M4 receptors in ME/CFS patients support their usage as clinical biomarkers in the diagnostic algorithm of ME/CFS.

## Data availability statement

The raw data supporting the conclusions of this article will be made available by the authors, without undue reservation.

## Ethics statement

The studies involving human participants were reviewed and approved by The study design was approved by the Ethical Committee of Rīga Stradiņš University (Ethical code Nr.6-1/05/33 and date of approval 30.04.2020.) and a written consent was obtained from all participants. The patients/participants provided their written informed consent to participate in this study.

## Author contributions

SG, YS, MM contributed to conception and design of the study. SG, AV, SR-D performed experiments. ZN-K, AK organized the database. SS performed the statistical analysis. SG, MM wrote the first draft of the manuscript. IL, KV wrote sections of the manuscript. All authors contributed to the article and approved the submitted version.

## Funding

This research was funded by EU H2020 project VirA, No.952376 and by the Latvian Science Council’s Fundamental and Applied Research project Nr. LZP-2019/1-0380.

## Conflict of interest

The authors declare that the research was conducted in the absence of any commercial or financial relationships that could be construed as a potential conflict of interest.

## Publisher’s note

All claims expressed in this article are solely those of the authors and do not necessarily represent those of their affiliated organizations, or those of the publisher, the editors and the reviewers. Any product that may be evaluated in this article, or claim that may be made by its manufacturer, is not guaranteed or endorsed by the publisher.
